# Thrifty phenotype versus cold adaptation: trade-offs in upper limb proportions of Himalayan populations of Nepal

**DOI:** 10.1098/rsos.172174

**Published:** 2018-06-20

**Authors:** Stephanie Payne, Rajendra Kumar BC, Emma Pomeroy, Alison Macintosh, Jay Stock

**Affiliations:** 1 Department of Archaeology, University of Cambridge, Cambridge, UK; 2 Pokhara University Research Centre, Pokhara, Nepal; 3 School of Natural Sciences and Psychology, Liverpool John Moores University, Liverpool, UK; 4 Department of Anthropology, University of Western Ontario, London, Ontario, Canada

**Keywords:** human adaptation, altitude, energetic stress

## Abstract

The multi-stress environment of high altitude has been associated with growth deficits in humans, particularly in zeugopod elements (forearm and lower leg). This is consistent with the thrifty phenotype hypothesis, which has been observed in Andeans, but has yet to be tested in other high-altitude populations. In Himalayan populations, other factors, such as cold stress, may shape limb proportions. The current study investigated whether relative upper limb proportions of Himalayan adults (*n* = 254) differ between highland and lowland populations, and whether cold adaptation or a thrifty phenotype mechanism may be acting here. Height, weight, humerus length, ulna length, hand length and hand width were measured using standard methods. Relative to height, total upper limb and ulna lengths were significantly shorter in highlanders compared with lowlanders in both sexes, while hand and humerus length were not. Hand width did not significantly differ between populations. These results support the thrifty phenotype hypothesis, as hand and humerus proportions are conserved at the expense of the ulna. The reduction in relative ulna length could be attributed to cold adaptation, but the lack of difference between populations in both hand length and width indicates that cold adaptation is not shaping hand proportions in this case.

## 1. Introduction

Life at high altitude is associated with extreme environmental stresses [1–8]. Hypoxia, low temperatures, a physically demanding lifestyle and nutritional constraints create a multi-stress environment which is inhospitable to longer term occupation by many human populations [8]. Populations who reside permanently in high-altitude regions have adapted to deal with the extreme stresses. Quantitatively different phenotypes have developed across the globe in high-altitude regions, demonstrating that multiple adaptive pathways have evolved to deal with high-altitude stresses [4–9] (table 1). Hypoxia is one of the few environmental stresses that cannot be effectively buffered by cultural adaptation [26], and so adaptive responses to hypoxia must occur through biological pathways to enable long-term survival of populations at high altitude [4,6,8]. High-altitude populations have evolved efficient mechanisms for dealing with hypoxia (table 1), but energetic deficits associated with life at high altitude often result in trade-offs during growth, creating a different phenotype from lowland populations [10,16,27–34].

**Table 1.** List of traits found in high-altitude populations (greater than 3000 m) compared with local lowland native groups. ↑ denotes increase; ↓, decrease; ↔, no difference.

	high-altitude region		
trait	Himalayas/Tibet	Andes	Ethiopia
height	↓ [9,10]	↓ [4]	↑ [11]
sitting height	↑ [12]	↑ [13]	↑ [14]
relative zeugopod length	↓ [15]	↓ [16]	↓ [14]
fat mass	↓ [17]	↓ [18]	↓ [11]
chest volume	↑ [12]	↑ [19]	↑ [11]
exhaled nitric oxide	↑ [20]	↑ [21]	↑ [22]
erythrocytosis	↔ [6]	↑ [6]	↔ [23]
arterial oxygen concentration	↓ [24]	↑ [24]	↔ [4]
altitude sickness with age	↑ [23]	↑ [25]	↔ [23]

### 1.1. Plastic growth

Linear growth during infancy and childhood appears to be moderately reduced with increasing altitude in Andean and Himalayan populations relative to their lowland counterparts [30,35–37], likely due to developmental plasticity. This height deficit has been commonly attributed to hypoxic stress, whereby limited oxygen compromises growth [30–33,38–42]. However, recent evidence suggests that oxygen saturation does not correlate with height in high-altitude Andean populations, indicating that nutrition and socioeconomic factors may play a more important role in stunted growth patterns [28,32]. Indeed, it is likely to be multiple high-altitude-related stresses contributing to reduced growth in high-altitude populations.

Clarifying where in the body the reduction in growth occurs is a strong indicator of the reason behind reduced height. The most significant decrement in height relative to lowland populations occurs in tibial growth, while sitting height remains the same [15,29]. The reduction in tibia length is mirrored by a reduction in radius length in some Andean populations [16], although this currently remains untested in Himalayan populations. This relative reduction in zeugopod length with altitude has been attributed to a thrifty phenotype mechanism [43], whereby exposure to environmental stress during early life can lead to growth trade-offs between different body elements. In an Andean population, autopod lengths (hands and feet) were seen to be conserved at the expense of other limb segments (forearm and lower leg) [16]. The authors argued that this pattern preserved function in the hands and feet, and that this pattern was inconsistent with the alternative distal blood flow hypothesis [44], which would predict a gradient of decreasing relative distal segment length with increased distance from the body as a result of progressively reduced nutrient availability. It remains untested whether the same pattern of relative size in different segments of the extremities is observed in high-altitude Himalayans. Greater cold stress in the Himalayas may result in different limb proportions from those of Andeans.

### 1.2. Potential cold adaptation

While both the Himalayas and the Andes have considerable local variation in temperature and humidity, high-altitude populations in the Himalayas are exposed to lower temperatures on average compared with Andeans due to differences in latitude, topography, rainfall and ecology [45]. The highland populations of Peru, Ecuador and Bolivia, residing up to as high as 4500 m above sea level, are likely to experience limited seasonality, but a significant range in diurnal temperature [46]. During winter, highlanders in cold, arid regions, such as Oruro and Bolivia, will tend to experience daily temperatures such as 5–10°C, with minimum temperatures dropping to approximately −10°C. Minimum temperatures are significantly lower in Himalayan settlements, reaching below −40°C in winter [47,48]. These lower temperatures may be greater selection pressures for good thermoregulation and minimizing risk of cold injury, and thus thermal selection pressures may have shaped the limb morphology of Himalayan populations unlike other high-altitude populations. Himalayan limb morphology may resemble the cold-adapted patterns found in other populations exposed to low temperatures [49], such as shorter and broader first metacarpals in individuals residing in cold climates than individuals from hot climates. This supports Allen's rule [50], where appendage length is reduced and appendage breadth increased to reduce heat loss in a cold climate.

Thus, applying Allen's rule to predict limb proportions in Himalayan populations, we would expect them to have shorter and broader limbs to minimize heat loss. Minimizing heat loss would reduce energetic demands on the body from maintaining body temperature, which may well be selected for as energetic stress is already strong in these populations as a result of multiple altitude-related stresses. Furthermore, low temperatures would also put individuals at greater risk of cold injury in the extremities [51,52]. Although there are individually reported cases of Sherpas with frostbite [51,53], they tend to have a lower incidence than recreational mountaineers [54,55]. These findings suggest that Sherpa hands may be better adapted to life in cold conditions, but whether hand dimensions play a role remains untested. By measuring hand dimensions of a sample of Sherpas, it may be possible to infer whether both their absolute and relative hand dimensions are suited to heat preservation or not.

As the extremity proportions of permanent Himalayan populations remain poorly documented [12,37,56], it is currently not possible to infer the key environmental stresses in Himalayan high-altitude upper limb morphology and how the trade-off is balanced between dexterity and thermoregulation. Thus, the current study investigates the limb proportions of highland and lowland groups from the Himalayas to determine how the multi-stress environment of high altitude influences limb morphology.

## 2. Material and methods

### 2.1. Study sample

The lowland population (*n* = 71) was sampled from a migrant Tibetan community in Jawalakhel, Kathmandu, Nepal (1400 m above sea level, 27.6744° N, 85.3123° E; average minimum winter temperature = 3.1°C [47]). This community was selected as they share common genetic ancestry with the highland population [57], and have similar diets and activity levels. The highland population (*n* = 183) was sampled from several Sherpa communities in Namche Bazaar and surrounding villages, Nepal (3500 m+ above sea level, 27.8069° N, 86.7140° E; average minimum winter temperature = −7.9°C [47]). Each participant self-identified as Tibetan and Sherpa in the lowland and highland populations, respectively, and evidence of birthplace was confirmed when possible through birth certificates or school records. A convenience sample of 254 participants between the age of 18 and 59 was measured.

### 2.2. Methods

Height was measured to the nearest millimetre using a Seca Leicester Height Measure following standard protocols with participants dressed in light clothing and unshod [3,4]. Body mass was measured to the nearest 0.05 kg using SECA-807 weighing scales (Seca, Birmingham, UK). Upper limb segment measurements were taken using a Trystom anthropometer a-226 (Trystom, spol s.r. o, Czech Republic). Both humerus and ulna lengths were measured following standard definitions [58]. Humerus length was measured from the lateral border of the acromion to the inferior extent of the olecranon (elbow flexed at 90°), while ulna length was taken from the olecranon to the head of the styloid process. Hand dimensions were measured following definitions by Davies *et al.* [59], with palm facing upwards, fingers and palm fully extended and hand flat, with dorsum of the hand resting on a horizontal surface. Hand length was measured from the level of the ulna styloid to the greatest extension of the middle finger perpendicular to the long axis of the hand. Hand width was measured as the linear distance between the radial side of the second metacarpophalangeal joint and the ulnar side of the fifth metacarpophalangeal joint. Humerus, ulna and hand lengths were summed to give total upper limb length.

### 2.3. Statistical analysis

To take account of differences in body size, upper limb segments relative to height were compared between populations. Relative segment lengths were calculated as follows:

$$\text{Relative segment length}=\frac{\text{Absolute segment length (cm) }}{\text{Height (cm) }}.$$

Both absolute and relative segment lengths were analysed using independent *t*-tests between the highland and lowland populations. To remove any sex differences, male and female data were analysed separately. Normality was tested using the Shapiro–Wilk test on all data. All statistical analysis was carried out using SPSS 25.0 for Windows.

## 3. Results

Absolute ulna length was significantly longer in lowlanders than in highlanders in both sexes (table 2). In males, highlanders were significantly shorter in height, total upper limb length, humerus length, ulna length and hand length. Absolute hand width did not significantly differ between populations in either sex.

**Table 2.** Descriptive statistics of highland and lowland populations. Sig., significance. Italics indicate statistically significant differences (*p* < 0.05).

	female			male		
	lowland (*n* = 42) mean (s.d.) (cm)	highland (*n* = 48) mean (s.d.) (cm)	sig.	lowland (*n* = 29) mean (s.d.) (cm)	highland (*n* = 135) mean (s.d.) (cm)	sig.
height	154.1 (±5.7)	155.5 (±6.3)	*p* > 0.05	168.2 (±7.0)	165.1 (±7.0)	*p* < *0.01*
total upper limb length	71.3 (±3.7)	70.0 (±3.3)	*p* > 0.05	77.6 (±3.9)	74.6 (±3.8)	*p* < *0.01*
humerus length	29.1 (±1.8)	29.0 (±2.0)	*p* > 0.05	31.4 (±1.8)	30.3 (±2.1)	*p* < *0.01*
ulna length	24.3 (±1.5)	23.2 (±1.4)	*p* < *0.01*	26.8 (±1.7)	25.4 (±1.8)	*p* < *0.01*
hand length	17.8 (±0.9)	17.7 (±0.9)	*p* > 0.05	19.4 (±1.3)	18.9 (±0.1)	*p* < *0.05*
hand width	9.2 (±0.5)	9.1 (±0.6)	*p* > 0.05	10.2 (±0.7)	9.9 (±0.6)	*p* > 0.05

**Figure 1.**
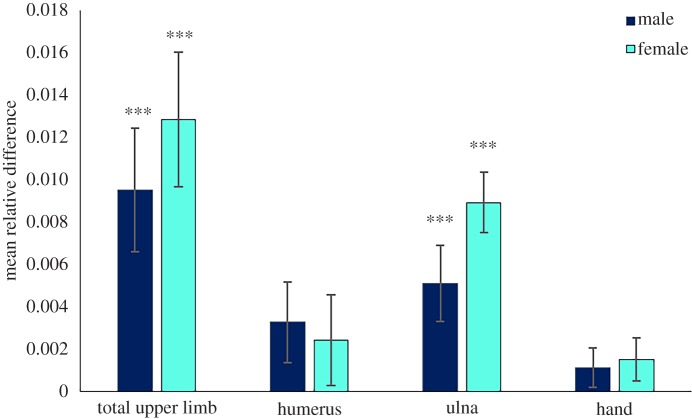
Bar chart of mean difference in upper limb segment length relative to height between lowland and highland populations (mean relative difference calculated as lowland relative mean minus highland relative mean); ***p < 0.01.

Relative to height, total upper limb and ulna lengths were significantly shorter in highlanders compared with lowlanders in both sexes, while relative hand length and width and relative humerus length were not significantly different between the two populations (figure 1: *p* > 0.05 for both sexes).

## 4. Discussion

These results are consistent with previous findings from Andean populations [16], as relative hand and humerus proportions are conserved at the expense of the ulna. This provides further support for a thrifty phenotype mechanism in shaping limb segment proportions in the presence of high-altitude stresses, and demonstrates that limb growth responds to environmental stress in Himalayan populations in a similar way to that seen in Andean populations. While the current study only investigated adults (aged 18–59), it indicates that the adult phenotype reflects the pattern which develops during childhood [16].

The current study aligns with prior evidence of selective growth under environmental stress [16,60]. No difference was found in relative hand length or width between the populations, indicating that no compromise in growth was made in hand dimensions. Relative ulna length was significantly shorter in highlanders relative to lowlanders, indicating reduced growth of this limb segment. Differences in altitude may result in this limb segment difference as limited oxygen availability may reduce growth in the highland population, as previously seen in other high-altitude populations [29,61]. However, this explanation is based on hypothetical assumptions relating to prioritization of functional elements and thus requires further investigation to fully understand the underlying mechanisms behind the limb segment pattern found here and elsewhere [10,15,35,62]

The reduction in relative ulna length could be attributed to cold adaptation [63,64], but the lack of difference between populations in both hand length and width indicates that cold adaptation is not shaping hand proportions in this case. It is possible that the forearms, but not hand proportions, are shaped by climate; Steegman [65] suggested that extreme vasoconstriction in the hands as a response to cold may negate any effect of hand proportions, as hand temperature may reach close to the surrounding temperature, and thus little heat is transferred to the surroundings from the hand. This is supported by cold immersion tests, whereby heat flux from the hand is consistently lower than heat flux from the forearm, even when a temporary cold-induced vasodilation response occurs in the fingers [66]. The forearm does not have such vasoregulatory responses, and thus maybe more susceptible to heat loss, and thus shortening of the zeugopod segment may have a significant effect on reducing energy expenditure via reduction in heat loss [65]. The mechanism for this adaptive limb segment shortening is unknown, but plasticity may play a role. It is well documented that temperature influences long bone elongation during postnatal development in several species, including mice [67–71], rats [72–74], rabbits [75] and pigs [76]. This plastic growth response to temperature may influence high-altitude long bone proportions; however, this plasticity in response to temperature has yet to be investigated in humans.

The hand proportions measured in the current study do not appear to align with cold adaptation theory. This may be for several reasons. Firstly, cold stress may not be the dominant factor influencing limb proportions; maintenance of hand dimensions for dexterity may be acting here [77]. Evidence in the skeletal record suggests that cold adaptation theory may explain patterns in hand proportions of high latitude-dwelling populations [49], but may not be applicable to high-altitude populations. The highland population in the current study may not show cold adaptation patterns in the hands as they may not be exposed to extreme low temperatures as regularly or for such prolonged periods as populations at very high latitudes and the high insolation of the Himalayas during the day may alleviate cold stress [47,48]. Alternatively, the results here may indicate that in Himalayan populations, temperature does not act on hand proportions through plastic mechanisms. As the lowland population had a shared genetic ancestry with the highland population [57], both populations may have the same genetic-based long-term adaptations which shape the hands, which may or may not relate to cold adaptation. Finally, there could be other modifying factors here, such as the use of gloves or insulative clothing in highlanders to alleviate any cold stress effects, but this was not measured in our study.

The results here do not support the distal blood flow hypothesis [44], as the hand was not significantly reduced in length or width relative to the rest of the body in highlanders compared with lowlanders. This again aligns with findings from Andean populations [16]. However, this limb proportion pattern may indirectly be linked to differential blood supply to hand and forearm segments. When blood vessels are fully perfused, blood supply is greater in autopod segments than zeugopod segments, due to dense capillary networks in the hands and feet [78], where blood moves slowly and thus nutrient delivery is highly efficient. Even if there is significant vasoconstriction in the highland populations during cold exposure, there may still be sufficient nutrient delivery to the deep tissue and bones of the hands, ensuring essential bone development and regeneration [79,80]. Whether vasoconstriction negates any effect of differential blood supply requires further investigation.

Although overall the diet and activity of the two populations were similar, there may have been some differences which were difficult to quantify. Lowland individuals self-reported a traditionally Tibetan diet, but may also have had access to Westernized food as globalization has increased the diversity of food products available in Kathmandu. Differences in activity may also have occurred; the women in both populations were homemakers and living relatively sedentary lifestyles; the men in the lowland population were factory workers, while the men in the highland population were porters. While the men in both populations were manual labourers, energy expenditure of activity was not directly measured in this case, so any differences in activity were unknown. Previous work indicates a very high daily energy expenditure of highland porters [81]; further investigation would be required to determine the daily energy expenditure of Jawalakhel factory workers.

The significant differences between males in all absolute variables other than hand width may be due to greater sensitivity to environmental stresses in males [82]. As five different variables show the same pattern between the male populations (height, total upper limb length, humerus length, ulna length and hand length), this is unlikely to be a chance outcome. Alternatively, confounding factors, such as unknown differences in diet or activity, as discussed above, may result in differences in body form between highland and lowland males. Although there is a discrepancy in sample size between males, there are no assumptions relating to sample size when applying the independent samples *t*-test, and thus differences in sample size should not have an effect. However, it is possible that the lack of differences identified in the female samples, other than the significant difference in relative ulna length, may result from a lack of power due to the relatively small sample sizes.

Although the absolute differences were greater in males, the differences in relative ulna length and total upper limb length were greater in females. This may indicate differential investment in segment lengths between the sexes during energetic stress, or alternatively, that the greater deficit in height in highland males reduces the relative differences in upper limb segment lengths. This outcome needs further investigation to determine why absolute differences between highland and lowland upper limb segment lengths are greater in males, but relative differences are greater in females.

## 5. Conclusion

The current study showed heterogeneous reductions in different upper limb segments in association with altitude-related stresses in Himalayan populations. Relative to height, total upper limb length was significantly shorter in highlanders than lowlanders, a difference driven largely by reduced ulna length. These results provide further support for the thrifty phenotype hypothesis, as hand dimensions are prioritized over other upper limb segments for their manipulative function. Cold adaptation patterns in the hand were not found in this study, indicating that other selection pressures dictate limb proportions in the Himalayan high-altitude environment.

## Supplementary Material

Supporting Dataset

## Data accessibility

The datasets supporting this article can be found at Dryad: http://dx.doi.org/10.5061/dryad.25p96 [84].

## References

[1] Morpurgo G, Arese P, Bosia A, Pescarmona G, Luzzana M, Modiano G 1976 Sherpas living permanently at high altitude: a new pattern of adaptation. Proc. Natl Acad. Sci. USA 73, 747–751. (doi:10.1073/pnas.73.3.747) 1062785 10.1073/pnas.73.3.747 PMC335995

[2] Moore L, Niermeyer S, Zamudio S 1998 Human adaptation to high altitude: regional and life-cycle perspectives. Am. J. Phys. Anthropol. 107(Suppl 27), 25–64. (doi:10.1002/(SICI)1096-8644(1998)107:27+<25::AID-AJPA3>3.0.CO;2-L) 9881522 10.1002/(sici)1096-8644(1998)107:27+<25::aid-ajpa3>3.0.co;2-l

[3] Moore L 2001 Human genetic adaptation to high altitude. High Alt. Med. Biol. 2, 257–279. (doi:10.1089/152702901750265341) 11443005 10.1089/152702901750265341

[4] Beall CM 2006 Andean, Tibetan, and Ethiopian patterns of adaptation to high-altitude hypoxia. Hum. Biol. 46, 18–24. (doi:10.1093/icb/icj004) 10.1093/icb/icj004 21672719

[5] West JB, Milledge JS, Schoene RB, Luks A 2013 High altitude medicine and physiology. Boca Raton, FL: Francis Group.

[6] Beall CM 2014 Adaptation to high altitude: phenotypes and genotypes. Annu. Rev. Anthropol. 43, 251–272. (doi:10.1146/annurev-anthro-102313-030000)

[7] Bigham AW, Lee FS 2014 Human high-altitude adaptation: forward genetics meets the HIF pathway. Genes Dev. 28, 2189–2204. (doi:10.1101/gad.250167.114) 25319824 10.1101/gad.250167.114 PMC4201282

[8] Gilbert-Kawai ET, Milledge JS, Grocott MPW, Martin DS 2014 King of the mountains: Tibetan and Sherpa physiological adaptations for life at high altitude. Physiology 29, 388–402. (doi:10.1152/physiol.00018.2014) 25362633 10.1152/physiol.00018.2014

[9] Pawson I 1976 Growth and development in high altitude populations: a review of Ethiopian, Peruvian and Nepalese studies. Proc. R. Soc. Lond. B 194, 83–98. (doi:10.1098/rspb.1976.0067) 11483 10.1098/rspb.1976.0067

[10] Pawson I 1977 Growth characteristics of populations of Tibetan origin in Nepal. Am. J. Phys. Anthropol. 47, 473–482. (doi:10.1002/ajpa.1330470320) 201173 10.1002/ajpa.1330470320

[11] Harrison GA et al. 1969 The effects of altitudinal variation in Ethiopian populations. Phil. Trans. R. Soc. Lond. B 256, 147–182. (doi:10.1098/rstb.1969.0040)

[12] Tripathy V, Gupta R 2007 Growth among Tibetans at high and low altitudes in India. Am. J. Hum. Biol. 19, 789–800. (doi:10.1002/ajhb.20638) 17691098 10.1002/ajhb.20638

[13] Trowbridge F, Marks J, Lopez de Romana G, Madrid S, Boutton T, Klein P 1987 Body composition of Peruvian children with short stature and high weight-for-height. II. Implications for the interpretation for weight-for-height as an indicator of nutritional status. Am. J. Clin. Nutr. 46, 411–418. (doi:10.1093/ajcn/46.3.411) 3630960 10.1093/ajcn/46.3.411

[14] Clegg E, Pawson I, Ashton E, Flinn R 1972 The growth of children at different altitudes in Ethiopia. Phil. Trans. R. Soc. Lond. B 264, 403–437. (doi:10.1098/rstb.1972.0015) 4404915 10.1098/rstb.1972.0015

[15] Bailey S, Hu X 2002 High-altitude growth differences among Chinese and Tibetan children. In Human growth from conception to maturity (eds Gilli G, Schell L, Benso L), pp. 237–247. London, UK: Smith-Gordon.

[16] Pomeroy E, Stock JT, Stanojevic S, Miranda JJ, Cole TJ, Wells JCK 2012 Trade-offs in relative limb length among Peruvian children: extending the thrifty phenotype hypothesis to limb proportions. PLoS ONE 7, e51795 (doi:10.1371/journal.pone.0051795) 23272169 10.1371/journal.pone.0051795 PMC3521697

[17] Boyer SJ, Blume FD 1984 Weight loss and changes in body composition at high altitude. J. Appl. Physiol. 57, 1580–1585. (doi:10.1152/jappl.1984.57.5.1580) 6520055 10.1152/jappl.1984.57.5.1580

[18] Haas J, Baker P, Hunt E 1977 The effects of high altitude on body size and composition of the newborn infant in Southern Peru. Hum. Biol. 49, 611–628. 590958

[19] Greksa L 1986 Chest morphology of young Bolivian high-altitude residents of European ancestry. Hum. Biol. 58, 427–443. 3733066

[20] Erzurum SC et al. 2007 Higher blood flow and circulating NO products offset high-altitude hypoxia among Tibetans. Proc. Natl Acad. Sci. USA 104, 17 593–17 598. (doi:10.1073/pnas.0707462104) 10.1073/pnas.0707462104 PMC2077056 17971439

[21] Beall C, Laskowski D, Strohl K, Soria R, Villena M, Vargas E 2001 Pulmonary nitric oxide in mountain dwellers. Nature 414, 411–412. (doi:10.1038/35106641) 11719794 10.1038/35106641

[22] Beall CM, Laskowski D, Erzurum SC 2012 Nitric oxide in adaptation to altitude. Free Radical Biol. Med. 52, 1123–1134. (doi:10.1016/j.freeradbiomed.2011.12.028) 22300645 10.1016/j.freeradbiomed.2011.12.028 PMC3295887

[23] Xing G et al. 2008 Adaptation and mal-adaptation to ambient hypoxia; Andean, Ethiopian and Himalayan patterns. PLoS ONE 3, e2342 (doi:10.1371/journal.pone.0002342) 18523639 10.1371/journal.pone.0002342 PMC2396283

[24] Beall CM et al. 1997 Ventilation and hypoxic ventilatory response of Tibetan and Aymara high altitude natives. Am. J. Phys. Anthropol. 104, 427–447. (doi:10.1002/(SICI)1096-8644(199712)104:4<427::AID-AJPA1>3.0.CO;2-P) 9453694 10.1002/(SICI)1096-8644(199712)104:4<427::AID-AJPA1>3.0.CO;2-P

[25] Beall C, Strohl K, Gothe B, Britenham G, Barragan M, Vargas E 1992 Respiratory and hematological adaptations of young and older Aymara men native to 3600M. Am. J. Hum. Biol. 4, 17–26. (doi:10.1002/ajhb.1310040105) 28524404 10.1002/ajhb.1310040105

[26] Beall CM, Jablonski N, Steegman A 2012 Human adaptation to climate: temperature, ultraviolet radiation, and altitude. In Human biology: an evolutionary and biocultural perspective (eds Stinson S, Bogin B, O'Rourke D), pp. 177–250. Hoboken, NJ: John Wiley & Sons, Inc.

[27] Smith C 1997 The effect of maternal nutritional variables on birthweight outcomes of infants born to Sherpa women at low and high altitudes in Nepal. Am. J. Hum. Biol. 763, 751–763. (doi:10.1002/(SICI)1520-6300(1997)9:6<751::AID-AJHB8>3.0.CO;2-U) 10.1002/(SICI)1520-6300(1997)9:6<751::AID-AJHB8>3.0.CO;2-U 28561390

[28] Weitz CA, Garruto RM, Chin C-TC, Liu JJ-C 2004 Morphological growth and thorax dimensions among Tibetan compared to Han children, adolescents and young adults born and raised at high altitude. Ann. Hum. Biol. 31, 292–310. (doi:10.1080/0301446042000196316) 15204346 10.1080/0301446042000196316

[29] Bailey S, Xu J, Feng J, Hu X, Zhang C, Qui S 2007 Tradeoffs between oxygen and energy in tibial growth. Am. J. Hum. Biol. 19, 662–668. (doi:10.1002/ajhb) 17636531 10.1002/ajhb.20667

[30] Argnani L, Cogo A, Gualdi-Russo E 2008 Growth and nutritional status of Tibetan children at high altitude. Coll. Antropol. 32, 807–812. 18982755

[31] Moore L, Charles S, Julian C 2011 Humans and high altitude: hypoxia and fetal growth. Physiol. Neurobiol. 178, 181–190. (doi:10.1016/j.resp.2011.04.017) 10.1016/j.resp.2011.04.017 PMC3146554 21536153

[32] Pomeroy E, Stock JT, Stanojevic S, Miranda JJ, Cole TJ, Wells JCK 2013 Associations between arterial oxygen saturation, body size and limb measurements among high-altitude Andean children. Am. J. Hum. Biol. 25, 629–636. (doi:10.1002/ajhb.22422) 23904412 10.1002/ajhb.22422 PMC3793237

[33] Pomeroy E, Stock JT, Stanojevic S, Miranda JJ, Cole TJ, Wells JCK 2014 Stunting, adiposity, and the individual-level ‘dual burden’ among urban lowland and rural highland Peruvian children. Am. J. Hum. Biol. 26, 481–490. (doi:10.1002/ajhb.22551) 24706334 10.1002/ajhb.22551 PMC4312888

[34] Weitz CA, Garruto RM 2015 Stunting and the prediction of lung volumes among Tibetan children and adolescents at high altitude. High Alt. Med. Biol. 16, 306–317. (doi:10.1089/ham.2015.0036) 26397381 10.1089/ham.2015.0036 PMC4685485

[35] Beall C 1981 Growth in a population of Tibetan origin at high altitude. Ann. Hum. Biol. 8, 31–38. (doi:10.1080/03014468100004761) 7224586 10.1080/03014468100004761

[36] Beall C 1984 Aging and growth at high altitudes in the Himalayas. In The people of south Asia (ed Beall C), pp. 365–385. New York, NY: Plenum Press.

[37] Gupta R, Basu A 1981 Variations in body dimensions in relation to altitude among the Sherpas of the eastern Himalayas. Ann. Hum. Biol. 8, 145–152. (doi:10.1080/03014468100004881) 7247343 10.1080/03014468100004881

[38] Bateson P et al. 2004 Developmental plasticity and human health. Nature 430, 419–421. (doi:10.1038/nature02725) 15269759 10.1038/nature02725

[39] Weinstein KJ 2005 Body proportions in ancient Andeans from high and low altitudes. 585, 569–585. (doi:10.1002/ajpa.20137) 10.1002/ajpa.20137 15895419

[40] Julian C, Vargas E, Armaza J, Wilson M, Niermeyer S, Moore L 2007 High-altitude ancestry protects against hypoxia-associated reduction in fetal growth. Arch. Dis. Child. Fetal Neonatal Ed. 92, F372–F377. 17329275 10.1136/adc.2006.109579 PMC2675361

[41] Eichstaedt C, Antao T, Cardona A, Pagani L, Kivisild T, Mormina M 2015 Genetic and phenotypic differentiation of an Andean intermediate altitude population. Physiol. Rep. 3, e12376 (doi:10.14814/phy2.12376) 25948820 10.14814/phy2.12376 PMC4463816

[42] Ge R-L, Simonson TS, Gordeuk V, Prchal JT, McClain DA 2015 Metabolic aspects of high-altitude adaptation in Tibetans. Exp. Physiol. 100, 1247–1255. (doi:10.1113/EP085292) 26053282 10.1113/EP085292 PMC10905973

[43] Hales CN, Barker DJP 1992 Type 2 (non-insulin-dependent) diabetes mellitus: the thrifty phenotype hypothesis. Int. J. Epidemiol. 42, 1215–1222. (doi:10.1093/ije/dyt133) 10.1093/ije/dyt133 24159065

[44] Lampl M, Kuzawa C, Jeanty P 2003 Prenatal smoke exposure alters growth in limb proportions and head shape in the midgestation human fetus. Am. J. Hum. Biol. 15, 533–546. (doi:10.1002/ajhb.10140) 12820195 10.1002/ajhb.10140

[45] Barry R 1992 Mountain weather and climate, 2nd edn. London, UK: Routledge.

[46] Thomas R, Winterhalder B 1976 Physical and biotic environment of southern highland Peru. In Man in the Andes: a multidisciplinary study of high-altitude Quechua (eds Baker P, Little M), pp. 46–58. Stroudsburg, PA: Dowden, Hutchinson and Ross, Inc.

[47] Merkel A 2016 Nepal — Climate Data. Climate-Data.org.

[48] Vuillermoz E 2016 Nepal Climate Observatory. *EVK2-CNR Everest Pyramid GAW Station* (accessed 6 May 2016).

[49] Betti L, Lycett SJ, Von Cramon-Taubadel N, Pearson OM 2015 Are human hands and feet affected by climate? A test of Allen's rule. Am. J. Phys. Anthropol. 158, 132–140. (doi:10.1002/ajpa.22774) 26119250 10.1002/ajpa.22774

[50] Allen J 1877 The influence of physical conditions on the genesis of species. Radic. Rev. 1, 108–140.

[51] Subedi BH, Pokharel J, Thapa R, Banskota N, Basnyat B 2010 Frostbite in a Sherpa. Wilderness Environ. Med. 21, 127–129. (doi:10.1016/j.wem.2009.12.031) 20591374 10.1016/j.wem.2009.12.031

[52] Moore GWK, Semple JL 2011 Freezing and frostbite on Mount Everest: new insights into wind chill and freezing times at extreme altitude. High Alt. Med. Biol. 12, 271–275. (doi:10.1089/ham.2011.0008) 21962071 10.1089/ham.2011.0008

[53] Macdonald EB, Shrestha S, Chhetri MK, Sherpa R, Sherpa DG, Murray K, Sanati KA 2015 Work-health needs of high-altitude mountain guides (Sherpas) in Nepal—a pilot study. Int. J. Occup. Saf. Ergon. 21, 9–14. (doi:10.1080/10803548.2015.1017945) 26327257 10.1080/10803548.2015.1017945

[54] Takeoka M, Yanagidaira Y, Sakai A, Asano K, Fujiwara T, Yanagisawa K 1993 Effects of high altitudes on finger cooling test in Japanese and Tibetans at Qinghai Plateau. Int. J. Biometeorol. 37, 27–31. (doi:10.1007/BF01212763) 8468096 10.1007/BF01212763

[55] Maley MJ, Eglin CM, House JR, Tipton MJ 2014 The effect of ethnicity on the vascular responses to cold exposure of the extremities. Eur. J. Appl. Physiol. 114, 2369–2379. (doi:10.1007/s00421-014-2962-2) 25081130 10.1007/s00421-014-2962-2

[56] Sloan A, Masali M 1978 Anthropometry of Sherpa men. Ann. Hum. Biol. 5, 453–458. (doi:10.1080/03014467800003101) 727703 10.1080/03014467800003101

[57] Bhandari S et al. 2015 Genetic evidence of a recent Tibetan ancestry to Sherpas in the Himalayan region. Sci. Rep. 5, 16249 (doi:10.1038/srep16249) 26538459 10.1038/srep16249 PMC4633682

[58] Lohman T, Roche A, Martorell R. 1988 Anthropometric standardization reference manual. Champaign, IL: Human Kinetics Books.

[59] Davies BT, Abada A, Benson K, Courtney A, Minto I 1980 A comparison of hand anthropometry of females in three ethnic groups. Ergonomics 23, 179–182. (doi:10.1080/00140138008924731) 7398617 10.1080/00140138008924731

[60] Bogin B, Smith P, Orden AB, Varela Silva MI, Loucky J 2002 Rapid change in height and body proportions of Maya American children. Am. J. Hum. Biol. 14, 753–761. (doi:10.1002/ajhb.10092) 12400036 10.1002/ajhb.10092

[61] Greksa L 1990 Developmental responses to high-altitude hypoxia in Bolivian children of European ancestry. Am. J. Hum. Biol. 2, 603–612. (doi:10.1002/ajhb.1310020604) 28520129 10.1002/ajhb.1310020604

[62] Singh ASP, Sidhu LS, Malhotra P, Zeitschrift S, Juni H, Singh BSP, Sidhu LS, Malhotra P 1986 Body morphology of high altitude Spitians of North West Himalayas. Z. Morphol. Anthropol. 2, 189–195. 3739412

[63] Ruff CB 1991 Climate and body shape in hominid evolution. J. Hum. Evol. 21, 81–105. (doi:10.1016/0047-2484(91)90001-C)

[64] Wilberfoss P 2012 Cold case: cold induced vasodilation response, and the origins of Polynesian body morphology as an adaptation to a cold environment. PhD thesis, University of Auckland, New Zealand.

[65] Steegmann AT 2007 Human cold adaptation: an unfinished agenda. Am. J. Hum. Biol. 19, 165–180. 17286254 10.1002/ajhb.20614

[66] Wang D, Zhang H, Arens E, Huizenga C 2007 Observations of upper-extremity skin temperature and corresponding overall-body thermal sensations and comfort. Build. Environ. 42, 3933–3943. (doi:10.1016/j.buildenv.2006.06.035)

[67] Sumner F 1909 Some effects of external conditions upon the white mouse. J. Exp. Zool. 7, 97–154. (doi:10.1002/jez.1400070105)

[68] Sundstroem ES 1922 The adaptation of albino mice to an artificially produced tropical climate. I. Effect of the various factors composing a tropical climate on growth and fertility in mice. Am. J. Physiol. 60, 397–415.

[69] Ogle C 1934 Climatic influence on the growth of the male albino mouse. Am. J. Physiol. 107, 635–640.

[70] Serrat MA, King D, Lovejoy CO 2008 Temperature regulates limb length in homeotherms by directly modulating cartilage growth. Proc. Natl Acad. Sci. USA 105, 19 348–19 353. (doi:10.1073/pnas.0803319105) 10.1073/pnas.0803319105 PMC2614764 19047632

[71] Serrat MA, Williams RM, Farnum CE 2009 Temperature alters solute transport in growth plate cartilage measured by in vivo multiphoton microscopy. J. Appl. Physiol. 106, 2016–2025. (doi:10.1152/japplphysiol.00295.2009) 19372302 10.1152/japplphysiol.00295.2009 PMC2692772

[72] Chevillard L, Portet R, Cadot M 1963 Growth rate of rats born and reared at 5 and 30 C. Fed. Proc. 22, 699–703. 14020710

[73] Lee MM, Chu PC, Chan HC 1969 Magnitude and pattern of compensatory growth in rats after cold exposure. J. Embryol. Exp. Morphol. 21, 407–416. 5798122

[74] Riesenfeld A 1973 The effect of extreme temperatures and starvation on the body proportions of the rat. Am. J. Phys. Anthropol. 39, 427–459. (doi:10.1002/ajpa.1330390311) 4753139 10.1002/ajpa.1330390311

[75] Ogle C 1933 Animal adaptation to environmental temperature conditions. Am. J. Physiol. 103, 606–628.

[76] Weaver ME, Ingram D 1969 Morphological changes in swine associated with environmental temperature. Ecology 50, 710–713. (doi:10.2307/1936264)

[77] Marzke MW, Marzke R 2000 Evolution of the human hand: approaches to acquiring, analysing and interpreting the anatomical evidence. J. Anat. 197, 121–140. (doi:10.1046/j.1469-7580.2000.19710121.x) 10999274 10.1046/j.1469-7580.2000.19710121.x PMC1468111

[78] Standring S 2008 Upper limb, 40th edn. Edinburgh, UK: Churchill Livingstone, Elsevier.

[79] Ramasamy SK et al. 2016 Blood flow controls bone vascular function and osteogenesis. Nat. Commun. 7, 13601 (doi:10.1038/ncomms13601) 27922003 10.1038/ncomms13601 PMC5150650

[80] Tomlinson RE, Silva MJ 2013 Skeletal blood flow in bone repair and maintenance. Bone Res. 1, 311–322. (doi:10.4248/BR201304002) 26273509 10.4248/BR201304002 PMC4472118

[81] Malville NJ, Byrnes WC, Lim HA, Basnyat R 2001 Commercial porters of eastern Nepal: health status, physical work capacity, and energy expenditure. Am. J. Hum. Biol. 56, 44–56. (doi:10.1002/1520-6300(200101/02)13:1<44::AID-AJHB1006>3.0.CO;2-D) 10.1002/1520-6300(200101/02)13:1<44::AID-AJHB1006>3.0.CO;2-D 11466966

[82] Stinson S 1985 Sex differences in environmental sensitivity during growth and development. Yearb. Phys. Anthropol. 28, 123–147. (doi:10.1002/ajpa.1330280507)

[83] World Medical Association. 2013 World Medical Association Declaration of Helsinki: ethical principles for medical research involving human subjects. J. Am. Med. Assoc. 310, 2191–2194. (doi:10.1001/jama.2013.281053) 10.1001/jama.2013.281053 24141714

[84] Payne S, Kumar BC R, Pomeroy E, Macintosh A, Stock J 2018 Data from: Thrifty phenotype versus cold adaptation: trade-offs in upper limb proportions of Himalayan populations of Nepal Dryad Digital Repository. (http://dx.doi.org/10.5061/dryad.25p96) 10.1098/rsos.172174 PMC6030304 30110416

